# GIS analysis of the relationship between PM2.5 and acute CVD and respiratory hospitalizations

**DOI:** 10.1093/eurpub/ckac131.245

**Published:** 2022-10-25

**Authors:** H Slachtova, H Tomaskova, P Polaufova, J Michalik, I Tomasek, A Splichalova

**Affiliations:** 1 Faculty of Medicine, University of Ostrava, Ostrava-Vítkovice, Czechia; 2 Centre for Epidemiological Research, Univesity of Ostrava, Ostrava-Vítkovice, Czechia; 3 Department of Occupational Medicine, Public Health Institute, Ostrava, Czechia; 4 Department of Laboratories, Public Health Institute, Ostrava, Czechia

## Abstract

**Background and aims:**

The PM air pollution is a serious concern in northern Moravia in the Czech Republic. The aim is to evaluate the risk of acute hospital admissions for cardiovascular and respiratory causes with the use of the Geographic information system (GIS).

**Methods:**

The data on acute hospital admissions for cardiovascular (I00-99 according to ICD-10) and respiratory (J00-99) causes was assigned based on the information on residence to 77 geographical units (601,299 inhabitants). The annual concentrations of PM2.5 in the period 2013-2019 were assigned to this units according to the respective concentration iso-shapes (step 2 μg.m-3, concentrations ≤29 to ≥ 38 μg.m-3). The Incidence Rate Ratio (IRR) and 95% confidence interval (CI) was calculated for each concentration category. The incidence in the first category with the lowest PM2.5 concentrations (≤29 μg.m-3) was chosen the reference category. The statistical analyses were performed using the SW STATA v.15.

**Results:**

About a half of population (56%) in the year 2013 belonged into the PM2.5 category 34-35 μg.m-3, 26 thousand of inhabitants (4%) live in the PM2.5 concentrations ≥38 μg.m-3. During the analysed period the average concentration values decreased from 30.8 to 21.4 μg.m-3. A statistically significant risk of the acute hospitalization for cardiovascular causes was identified in the categories ≤36 μg.m-3, in the highest interval of PM2.5 the IRR values were 2-3-fold higher comparing with the reference category. As for respiratory causes, the trend is similar, but the statistically significant risk was found already from the interval 34-35 μg.m-3.

**Conclusions:**

With increasing concentrations, the risk of both acute cardiovascular, and respiratory hospitalizations increased.

This presentation was supported by the project TH03030195 of the Technology Agency of the Czech Republic and the project Healthy Aging in the Industrial Environment CZ.02.1.01/0.0/0.0/16_019/0000798 (HAIE).

**Key messages:**

• A statistically significant increase of the IRR for acute cardiovascular and respiratory hospitalizations was found at PM2.5 concentrations ≤34 μg.m-3 compared to the reference category ≤29 μg.m-3.

• Average annual PM2.5 concentration decreased from 30.8 to 21.4 μg.m-3 during the followed period and also the risk of acute hospitalization from cardiovascular and respiratory causes decreased.

